# Supplementary material for: Niche-specific metabolic phenotypes can be used to identify antimicrobial targets in pathogens
Source: PLoS Biol. 2024 Nov 18;22(11):e3002907. doi: 10.1371/journal.pbio.3002907 (PMC11611258; doi:10.1371/journal.pbio.3002907)
Supplement: S1 Text — This document includes 2 additional analyses to support the idea that the 80% completeness threshold does not impact our gene essentiality predictions. The data underlying Figs 1 and 2 is S1 Text can be found in Data1.zip. (PDF) [file pbio.3002907.s001.pdf]

***Completeness cutoff does not impact essentiality predictions***

We used the presence of 16S sequences as a surrogate for "more complete" genome sequences. We identified 367 sequences out of the 914 total sequences that contained 16s rRNA annotations, each from a unique strain in the collection of GENREs (still representing the same 9 phyla represented in all 914 models). Firstly, we wanted to ensure that the taxa included in the "more complete" subset of 367 sequences is representative of the whole set of 914 GENREs. To do this, we generated a cladogram of the 367 species with annotated 16s rRNA sequences (Figure 1 below). We determined the difference in the underlying topologies of the trees by calculating the number of splits present in one tree, but not the other by using the Robinson-Foulds method in the ete3 python toolkit. Our resulting Robinson-Foulds metric was 1, indicating that the underlying topologies of the 367 strain tree and the 914 strain tree are nearly identical. For context, a Robinson-Foulds distance of 0 would indicate identical topologies and the maximum Robinson-Foulds distance achievable in this scenario could have been 193. This analysis ensures that the members included in the 367 GENRE subset are representative of the whole collection.

While the cladogram analysis showed that both groups of genomes had similar coverage of microbial taxa, we performed an additional analysis with this revision to show that the functional predictions from both "more" and "less" complete genomes are also highly similar, suggesting that this completeness feature does not change the key messages from our analysis. To ensure that the completeness cutoff does not affect our resulting essentiality prediction, we generated essential gene profiles for each of the subset of 367 GENREs ("more" complete) as well as essential gene profiles for the entire collection of 914 GENREs were generated using an FBA-based, single-gene-knockout method in COBRApy

(cobra.flux_analysis.variability.find_essential_genes()). Simulations were used in a complete media context with open exchange reactions, resulting in a minimum number of essential genes. Essential genes were then converted to KEGG Orthologs. Figure 2 below is a histogram of the number of GENREs with a given essential gene across the subset of GENREs that are “more” complete (367 GENREs) and a histogram for essential genes across all GENREs (914 GENREs). Both are normalized to the number of members in the category (367/914). The similarity of the histograms below in Figure 2 (a) and (b) suggest that the essential genes identified across the whole collection (914) is representative of the essential genes identified in more complete sequences (367).

Secondly, for each essential gene, we calculated the difference between the percentage of GENREs containing a given essential gene in the “more complete” collection (367) compared to the total collection (914). The average difference in essential gene presence between the whole collection and the more “complete” subset is 1.3%. This result suggests that the essential genes identified in the entire collection of 914 GENREs (with “more” and “less” complete genomes) is representative of the more complete subset of GENREs (the 367 genomes that contain 16S sequences). This analysis helps to illustrate that the essential genes identified in the whole collection are similar to a subset with more complete genome sequences.

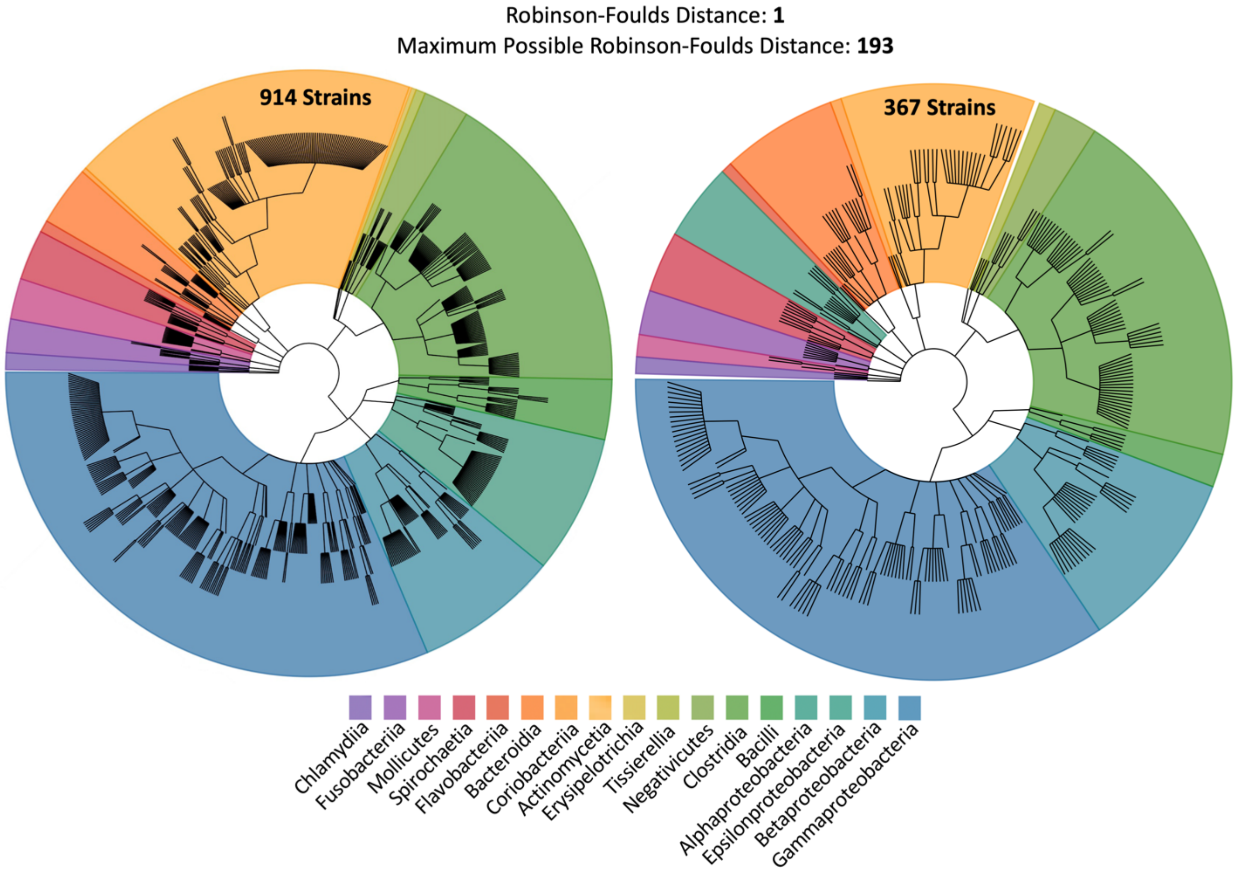


**Figure 1 | Cladogram comparison.** Cladogram of all 914 strains included in the GENRE (left), cladogram of 367 more 'complete' strains that contain 16S sequences. Reported Robinson-Foulds distance is 1 indicating similar underlying tree topologies.

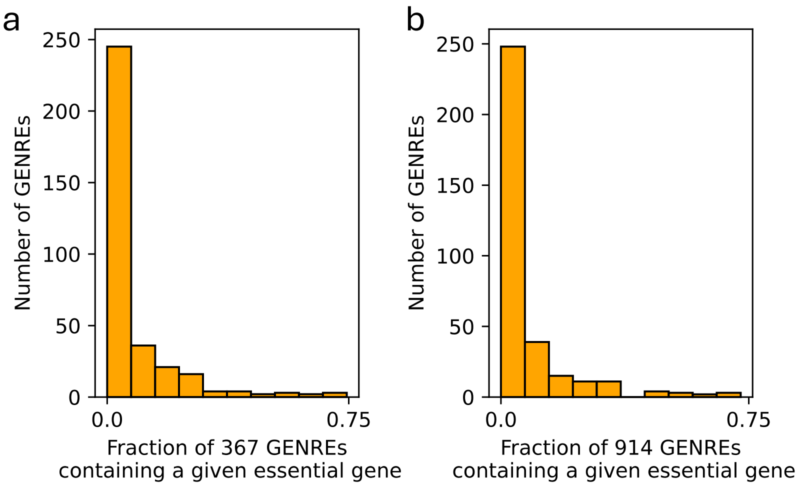


**Figure 2 | Essential gene predictions across all 914 GENREs and subset of 367 with higher-quality sequences.** a) Histogram of percent of GENREs containing a given essential gene across 367 GENRE subset. b) Histogram of percent of GENREs containing a given essential gene across 914 GENRE subset.
