# Supplementary figures and images for: Niche-specific metabolic phenotypes can be used to identify antimicrobial targets in pathogens
Source: PLoS Biol. 2024 Nov 18;22(11):e3002907. doi: 10.1371/journal.pbio.3002907 (PMC11611258; doi:10.1371/journal.pbio.3002907)

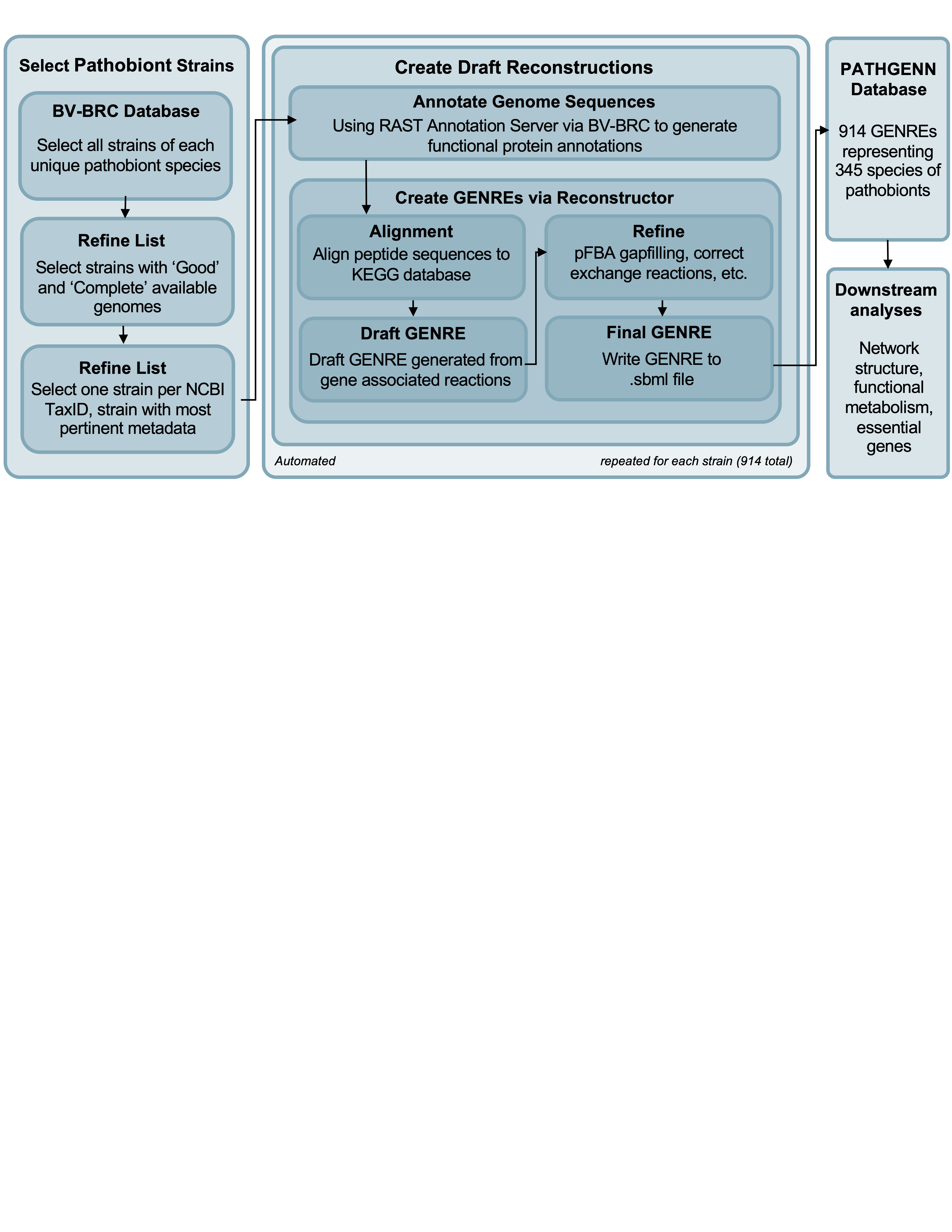

Supplement: S1 Fig — The BV-BRC database was used to select pathogen genome strains that satisfied quality criteria. These genome strains were then annotated using the RAST annotation toolbox to generate the amino acid FASTA file that was then used in Reconstructor to generate the 914 GENREs in the collection. (PNG) [file pbio.3002907.s002.png]

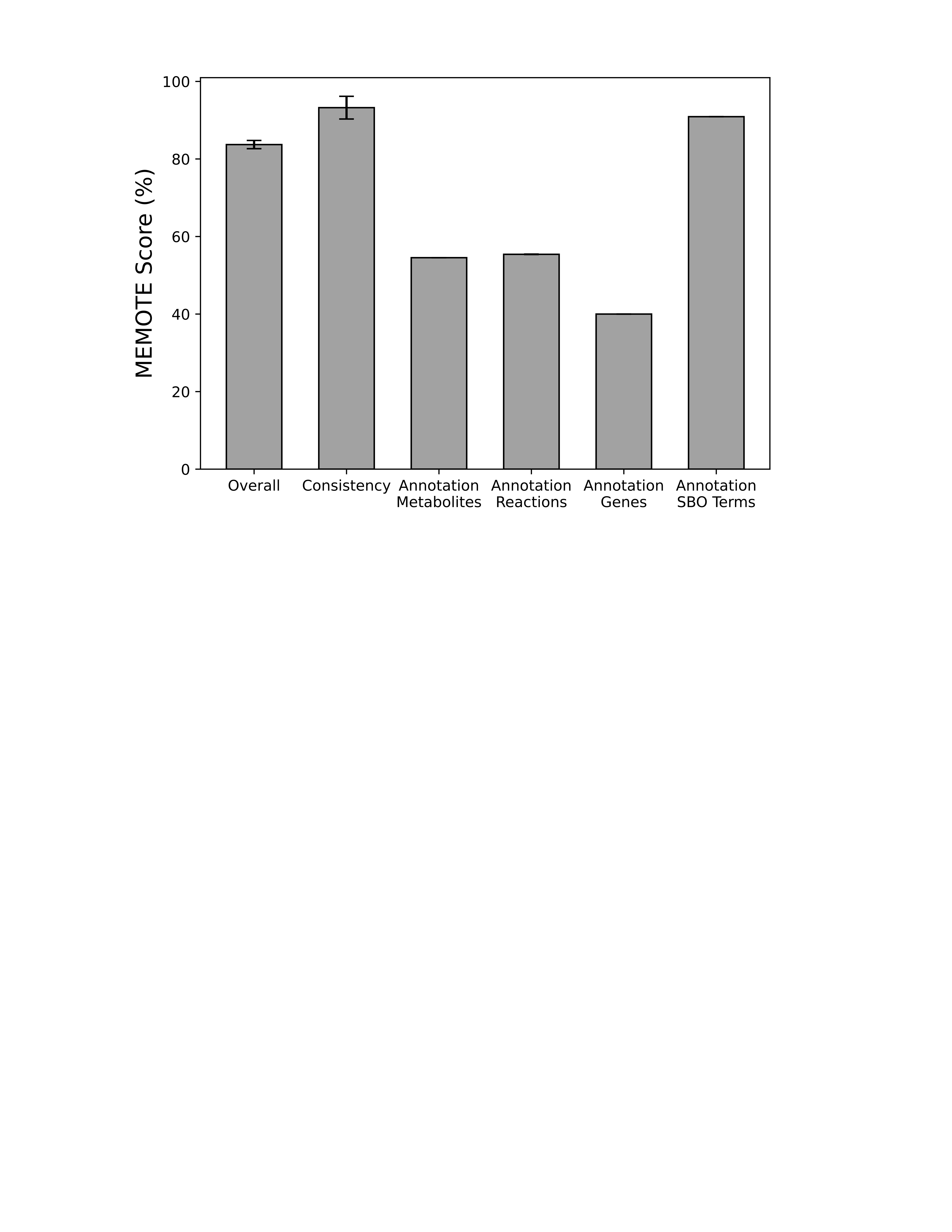

Supplement: S2 Fig — Overall MEMOTE scores had an average of 84%; subcategory scores were also considerably high with minimal variability in quality. The data underlying S2 Fig can be found in Data2Data3.csv on Zenodo: https://zenodo.org/records/13952471. (PNG) [file pbio.3002907.s003.png]

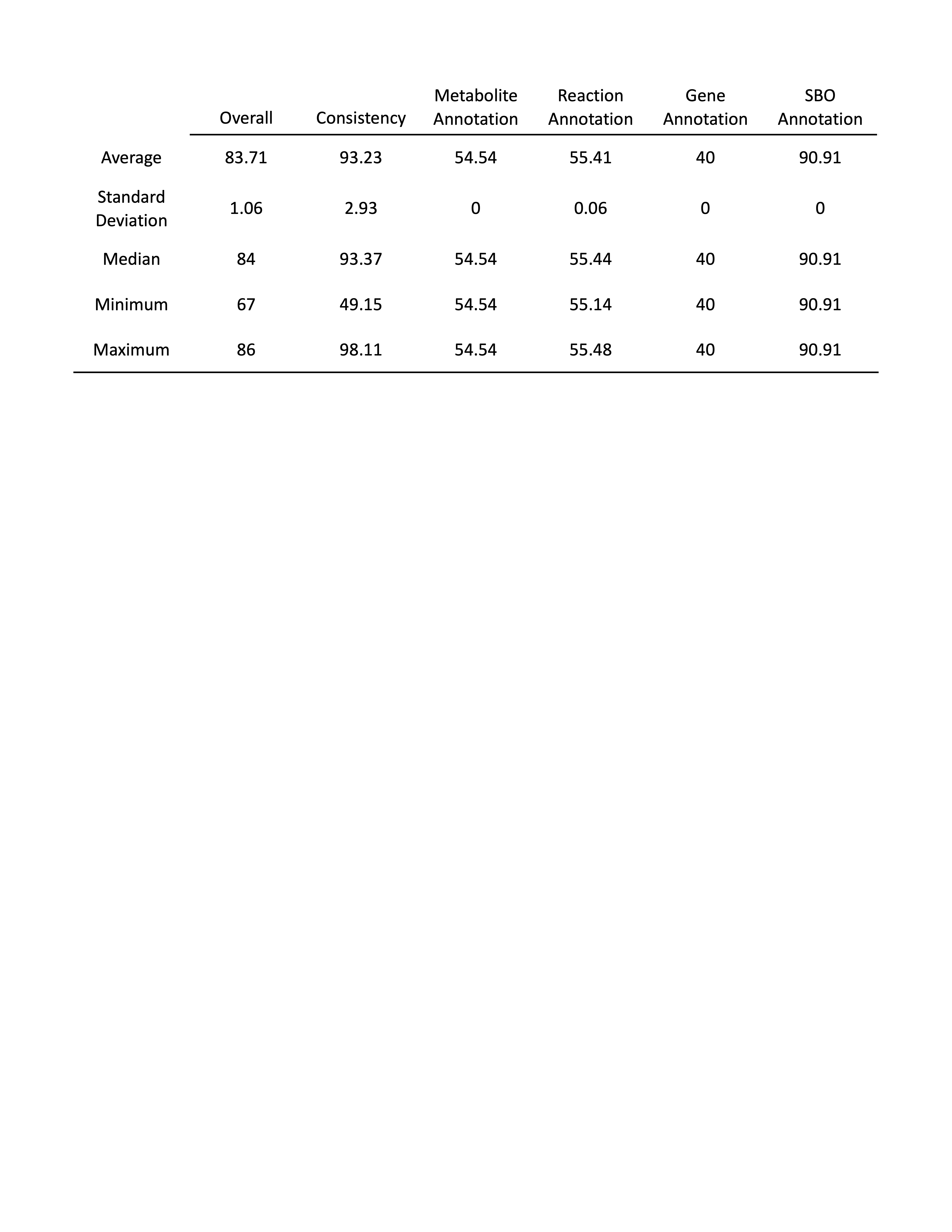

Supplement: S3 Fig — Average, standard deviation, median, minimum, and maximum reported for overall MEMOTE score as well as the consistency, metabolite annotation, reaction annotation, gene annotation, and SBO annotation subcategories. The data underlying S3 Fig can be found in Data2Data3.csv on Zenodo at DOI: https://zenodo.org/records/13952471. (PNG) [file pbio.3002907.s004.png]

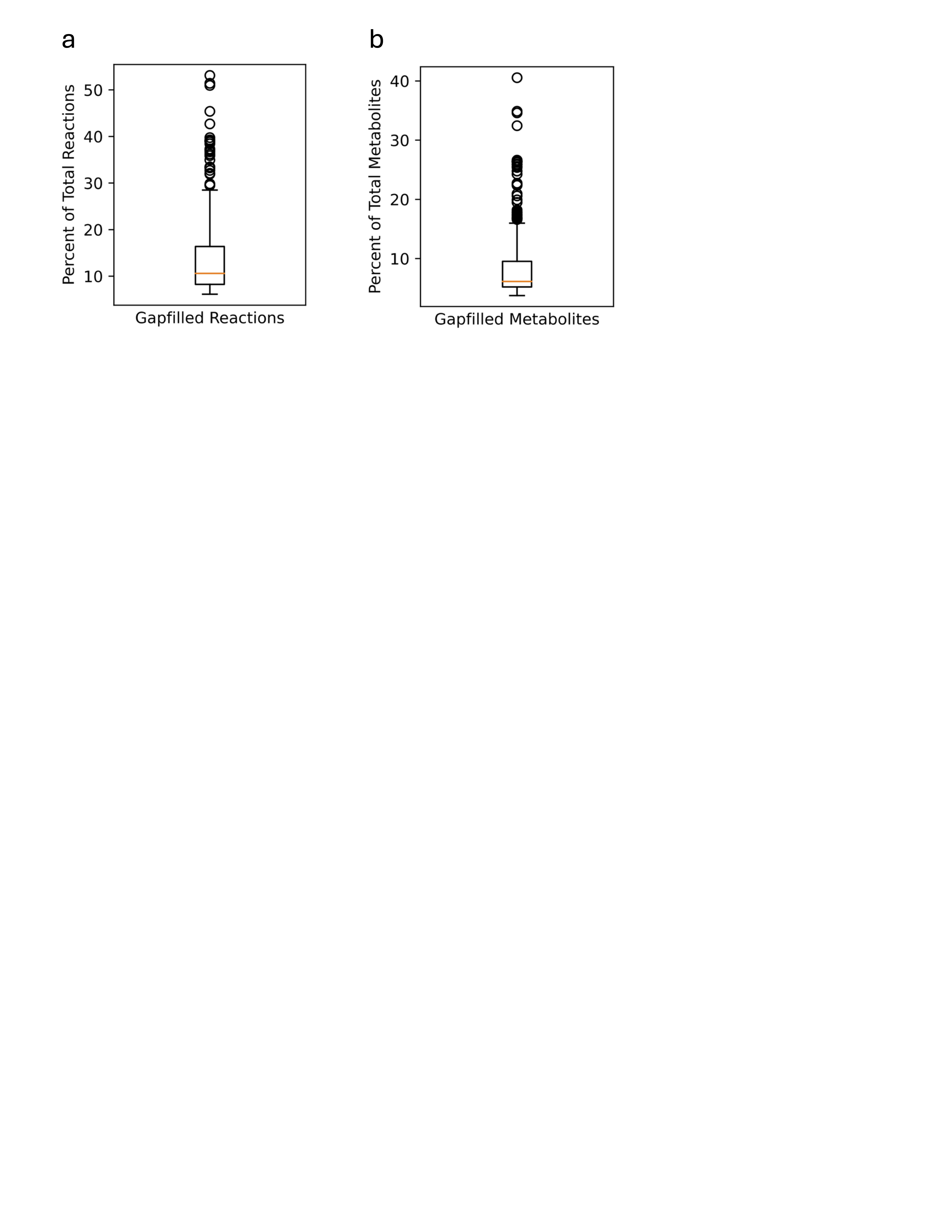

Supplement: S4 Fig — (a) Percent of total reactions gapfilled, (b) percent of total metabolites gapfilled. The data underlying S4 Fig can be found in Data4.xlsx on Zenodo: https://zenodo.org/records/13952471. (PNG) [file pbio.3002907.s005.png]

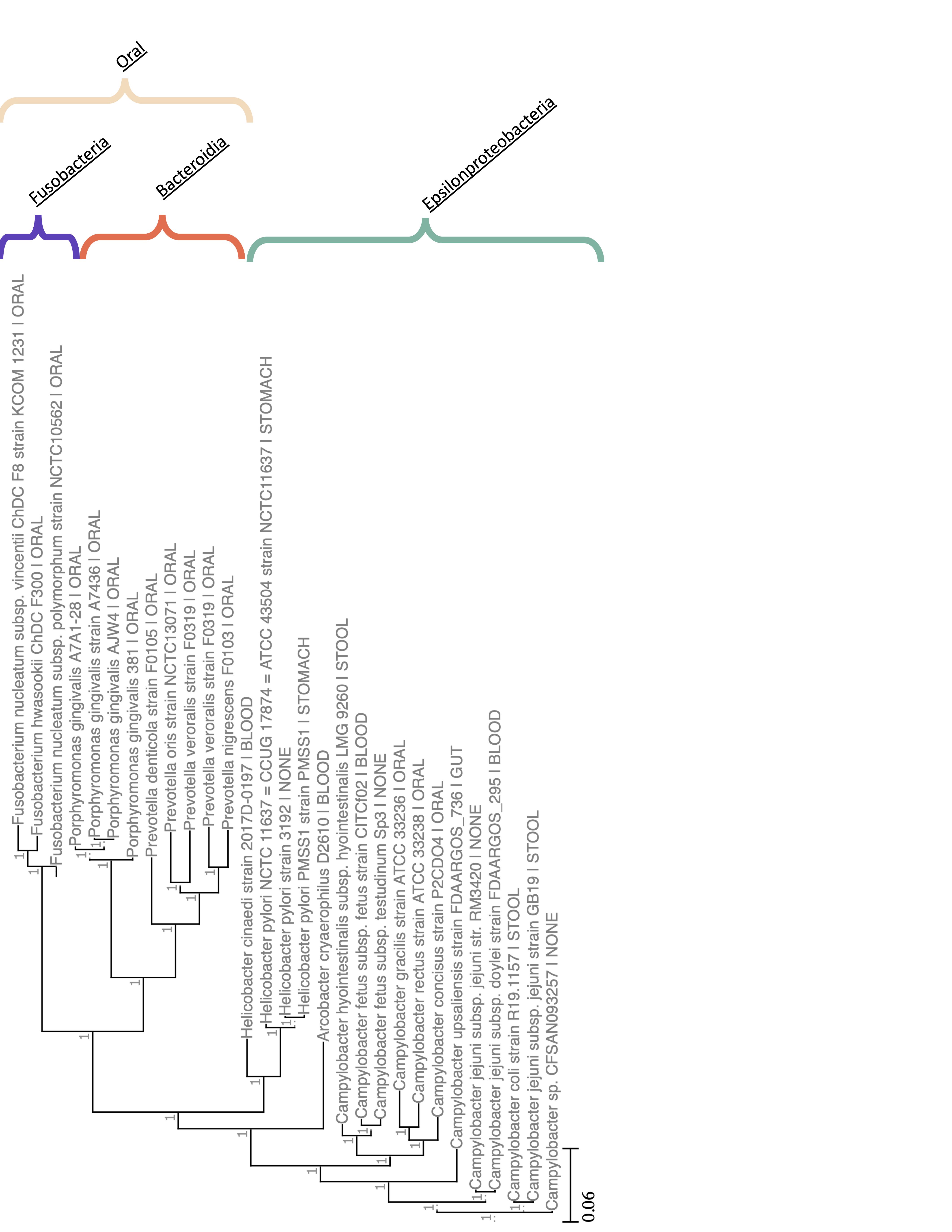

Supplement: S5 Fig — Fusobacteria and Bacteroidia species in the oral environment are not genetically similar. Epsilonproteobacteria are genetically similar, but occupy distinct environments. The data underlying S5 Fig can be found in Data5.txt on Zenodo: https://zenodo.org/records/13952471. (PNG) [file pbio.3002907.s006.png]

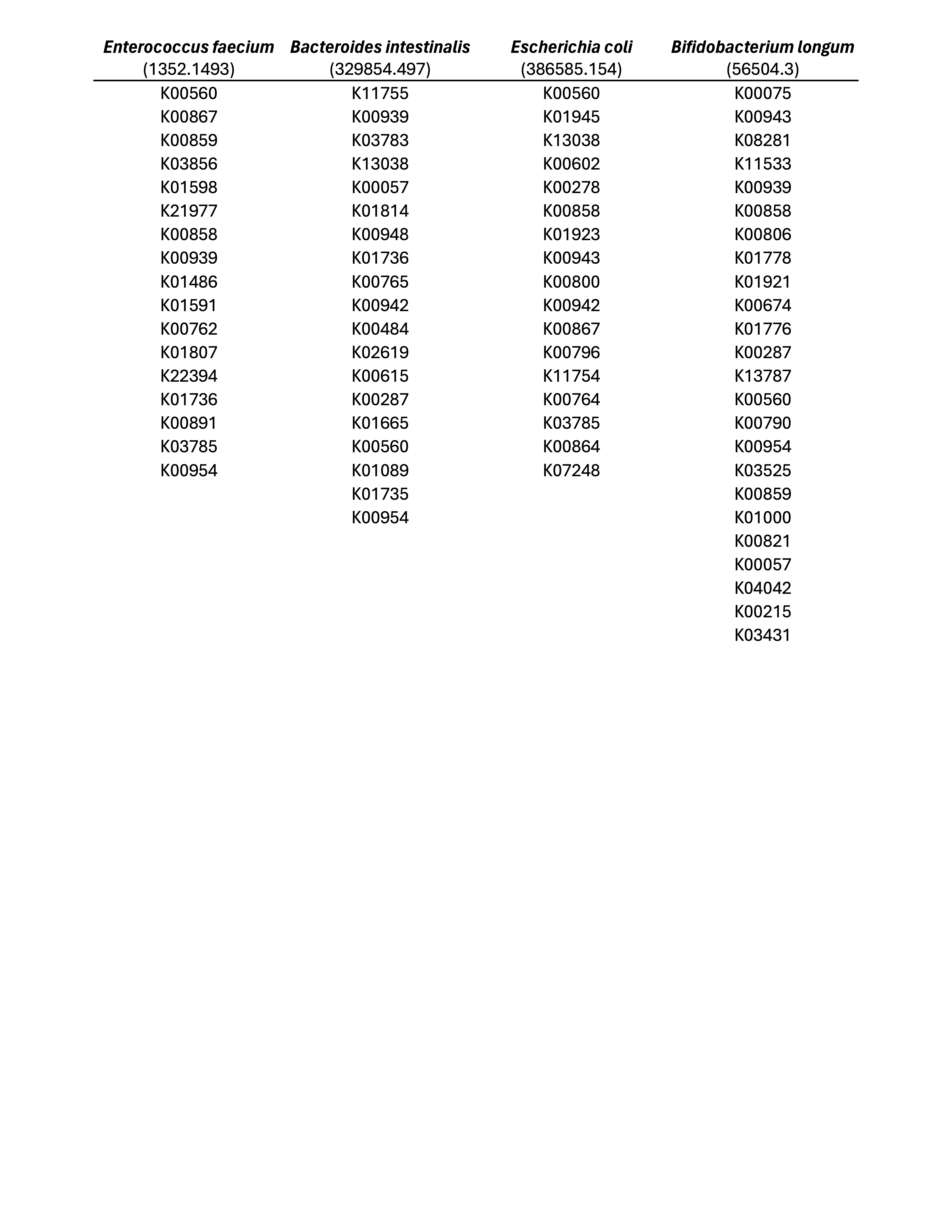

Supplement: S6 Fig — List of essential genes in 4 commensal bacterial species (listed by species name and BV-BRC genome ID). Essential genes are reported in KEGG identifiers. (PNG) [file pbio.3002907.s007.png]

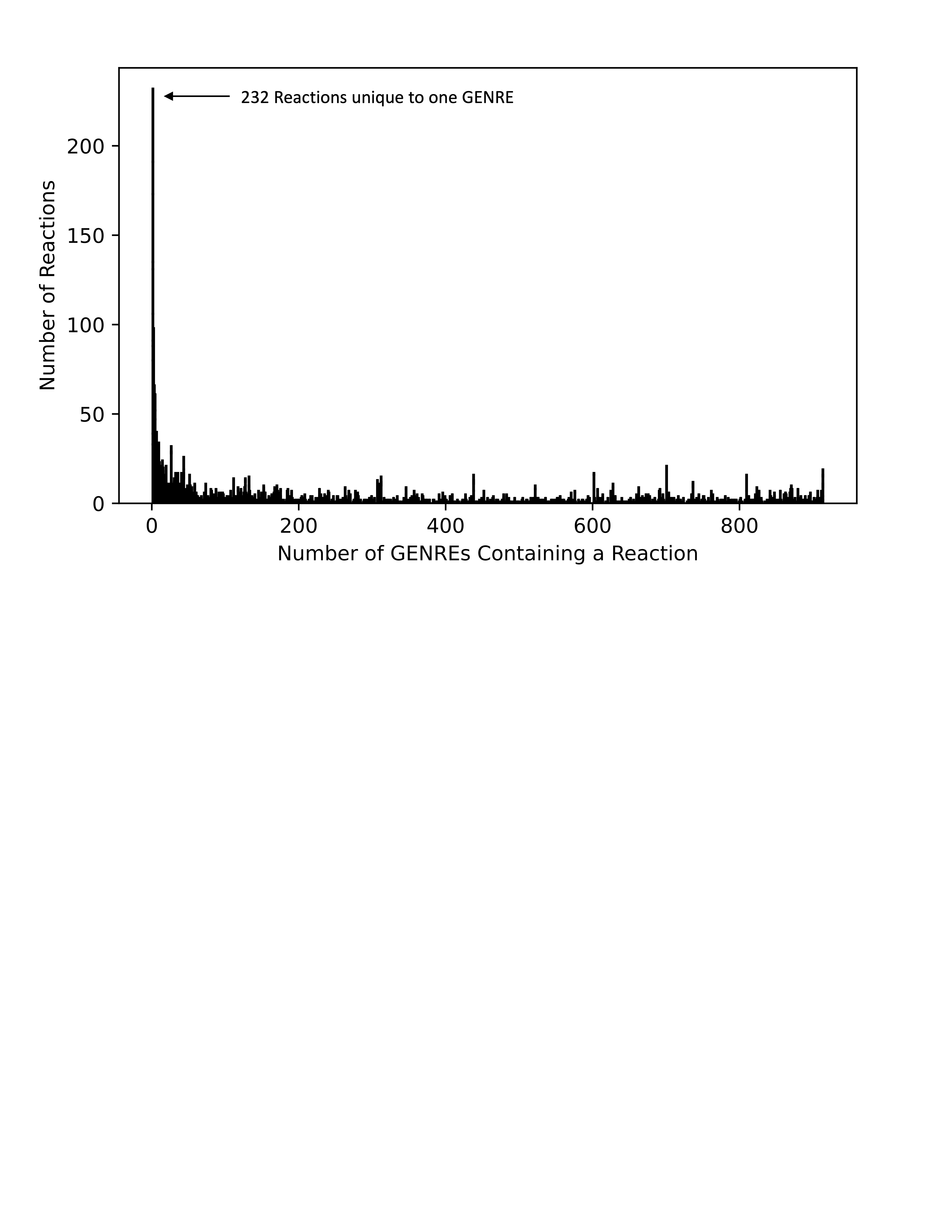

Supplement: S7 Fig — This plot is supplemental to the data presented in Fig 2A, using more stringent unique reaction cutoffs. The histogram has 914 bins which allow us to see that 232 reactions are unique to one GENRE. The data underlying S7 Fig can be found in Data7.csv on Zenodo: https://zenodo.org/records/13952471. (PNG) [file pbio.3002907.s008.png]

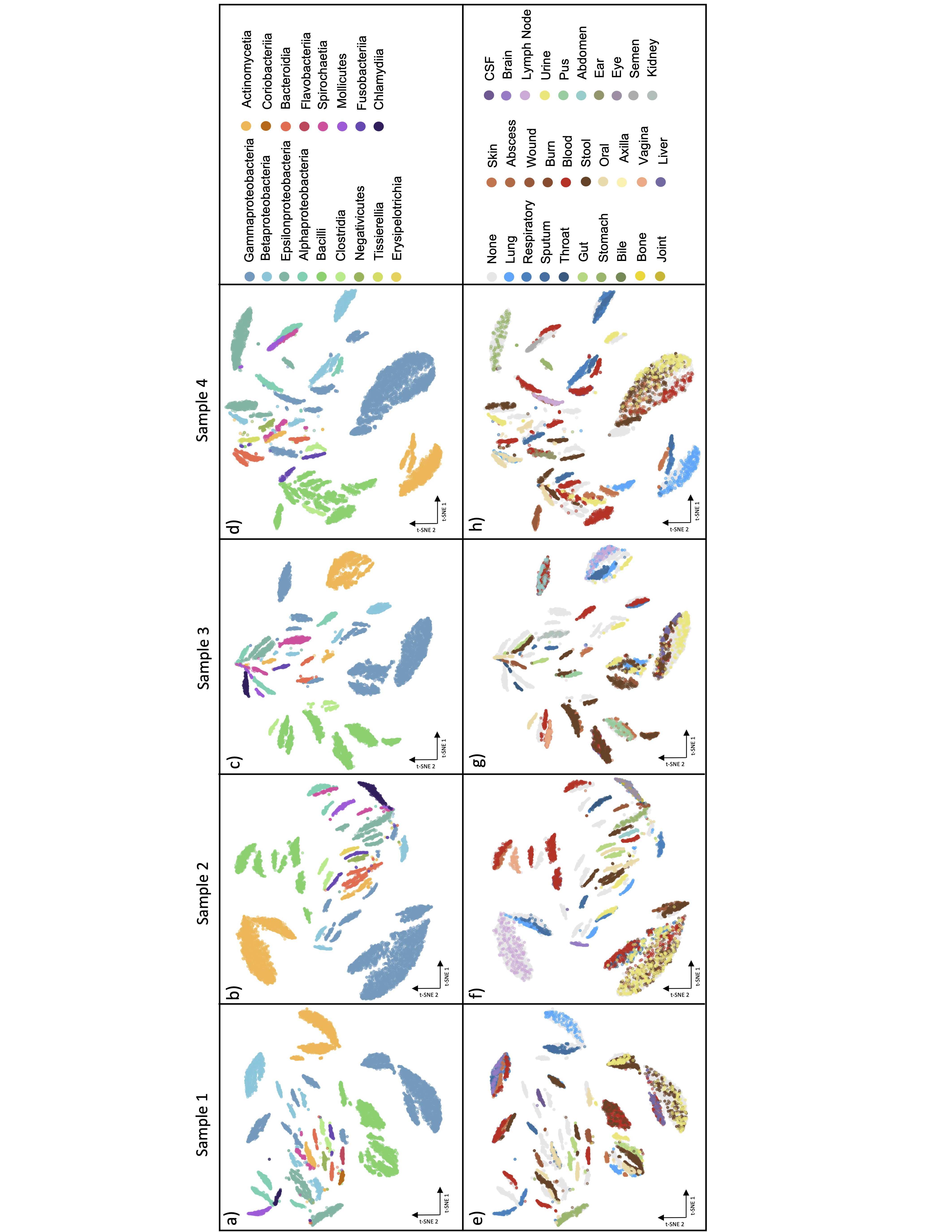

Supplement: S8 Fig — The clustering relationships seen in Fig 4 with 10 flux samples for each of 914 models are consistent with the clusters seen here with 3 randomly selected subsets of 100 GENREs with 100 flux samples each. Each pair of plots (a and e, b and f, c and g, d and h) represents randomly selected subset of 100 GENREs; (a–d) are colored based on taxonomic class; (e–h) are colored based on physiological location. The data underlying S8 Fig can be found in FluxSampleData.zip on Zenodo: https://zenodo.org/records/13952471. (PNG) [file pbio.3002907.s009.png]

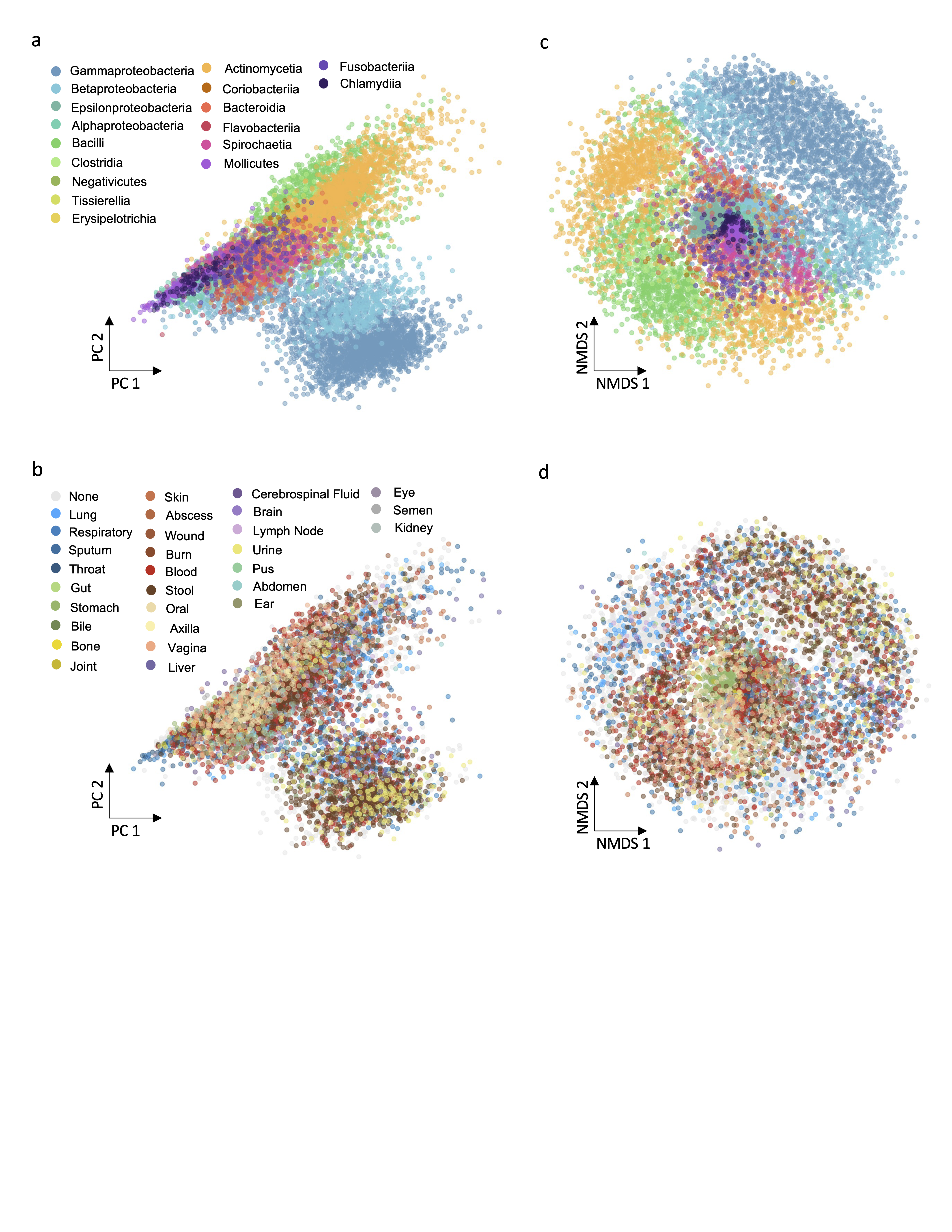

Supplement: S9 Fig — (a) principal component analysis, colored on taxonomic class. (b) Non-metric multidimensional scaling colored on taxonomic class. (c) Principal component analysis colored on isolate physiological location. (d) Non-metric multidimensional scaling. The data underlying S9 Fig can be found in FluxSampleData.zip on Zenodo: https://zenodo.org/records/13952471. (PNG) [file pbio.3002907.s010.png]

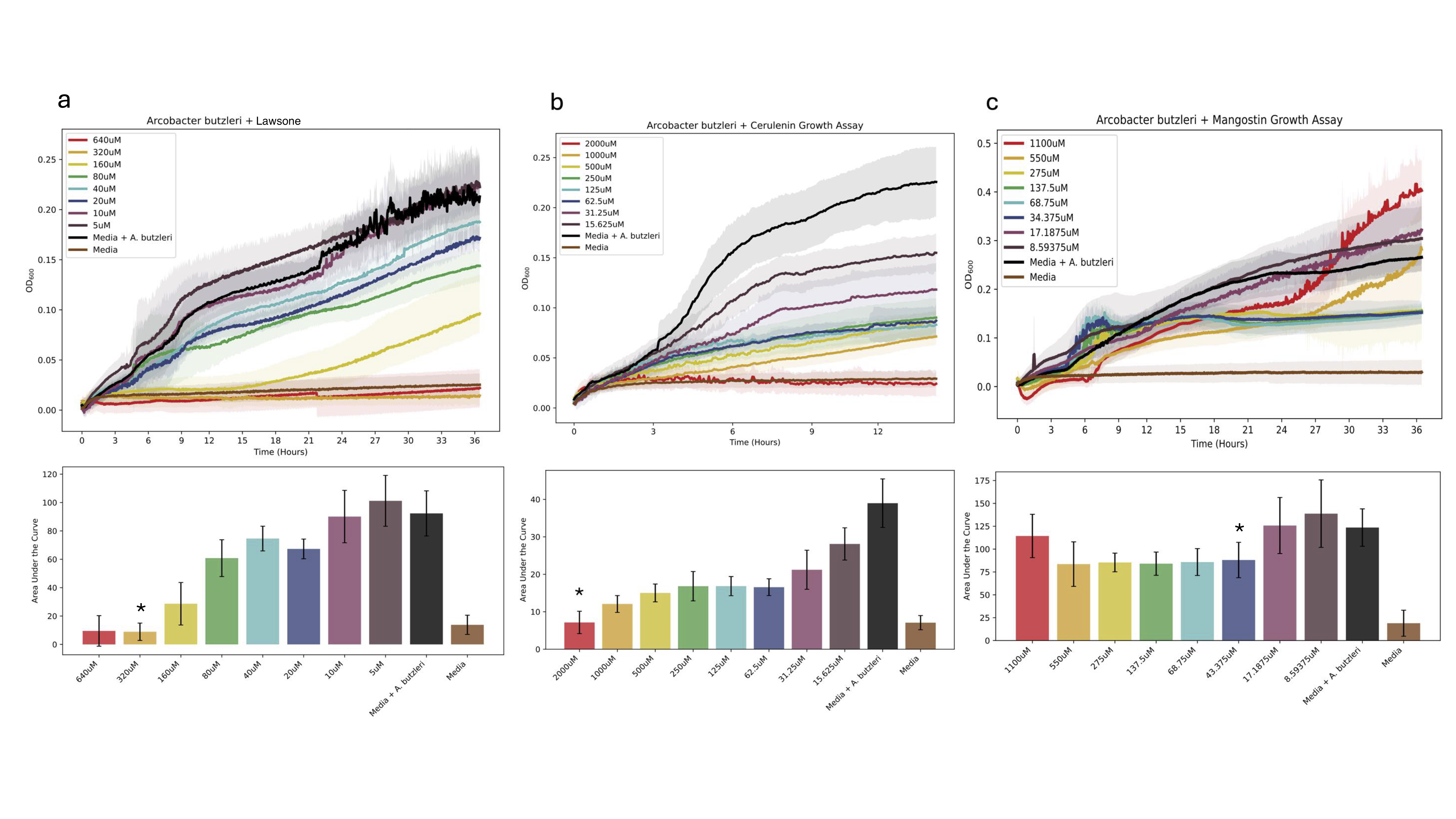

Supplement: S10 Fig — MIC assay with Arcobacter butzleri for each chemical inhibitor. Stars indicate the selected MIC, the concentration used in the subsequent validation experiments. The data underlying S10 Fig can be found in Data10.zip on Zenodo: https://zenodo.org/records/13952471. (PNG) [file pbio.3002907.s011.png]
