# Supplementary material for: Niche-specific metabolic phenotypes can be used to identify antimicrobial targets in pathogens
Source: PLoS Biol. 2024 Nov 18;22(11):e3002907. doi: 10.1371/journal.pbio.3002907 (PMC11611258; doi:10.1371/journal.pbio.3002907)
Supplement: Attachment — Submitted filename: ResponseToReviewers.pdf [file pbio.3002907.s012.pdf]

We appreciate the critiques of the reviewers. We have performed new analyses, updated our results with newer versions of the software tools, edited much of the text for clarity, and cleaned up the presentation of some of our previous analyses. These revisions and our efforts to respond to the critiques have resulted in a much improved manuscript. Below are the reviewer comments in **black** and our responses in **blue**.

REVIEWERS' COMMENTS:

Reviewer #1:

The manuscript by Glass et al. reports am interesting approach to antimicrobial target identification using genome-scale metabolic modelling. The central idea is to identify essential genes in a group of microbes that occupy the same "niche" under the infection context. Metabolic modelling provides an in-silico screen and enumeration of essential genes, with a subset of results validated in vitro using known small-molecule inhibitors. Overall, the manuscript is a valuable addition as a resource of metabolic models of pathogens with a proof-of-concept analysis showing how the resource could be used. There are, however, several points where the study is unclear / falls short of providing sufficient evidence to the conclusions. These are listed below, and I hope that these will be useful for the authors to improve the study/presentation.

We thank the reviewer for the favorable consideration of the manuscript and appreciate the constructive criticisms below.

1*. Genome selection for reconstruction: 80% completeness and 20% contamination are too loose cut-offs as these can introduce both type I and type II errors.*

We would like the reviewer to note that the contamination cutoff is defined in the manuscript as 10%, rather than 20%. Nonetheless, we acknowledge that these cutoffs for completeness and contaminations are relatively loose and could introduce false positives or negatives in our downstream computational analyses. We chose these cutoffs since these are how a “good” quality genome is defined in the BV-BRC database, and selecting a “good” quality genome is just a checkbox in the BV-BRC web browser. We do not have a readily accessible way to alter these criteria to be more stringent and select genomes with different completeness/contamination percentages.

“This is especially critical since the subsequent analysis is categorical, based on presence/absence of genes (and mapped reactions thereby).”

We acknowledge that the 80% and 10% completeness and contamination cutoffs could have implications when predicting essential genes in our downstream computational analyses. However, we have taken caution when making our essential gene calls to not make the cutoffs too stringent, because we recognize there are possible errors

introduced in several stages of model building, including this step of genome selection. For example, when identifying genes that are uniquely essential to stomach pathogens, we define a uniquely essential gene as “a gene that is essential in at least 80% of stomach associated isolates, and is not essential in at least 80% of isolates in all other physiological locations”. By instantiating this 80% cutoff rather than 100%, we are partially mitigate errors made in earlier model-building steps.

The importance of this point is highlighted by the fact that a vast number of the genomes did not have 16S regions.

We thank the reviewer for bringing this point to our attention. We understand how the strains missing the 16s annotations could be cause for concern, especially in the analysis where we rely on the 16s sequences for determining pairwise genetic similarity (Figure 2c). However, we took steps to ensure that the 367 strains that *did* have 16s sequences present were representative of the whole 914 strain collection. We first generated a phylogenetic tree to ensure that there was visual concordance between the distribution of taxonomic classes represented in the 367 strains compared to the 914 strain collection. We now include this cladogram in S9. Secondly, we determined the difference in the underlying topologies of the trees by calculating the number of splits present in one tree but not the other by using the Robinson-Foulds method in the ete3 tookit in Python. Our resulting Robinson-Foulds metric was 1, indicating that the underlying topology of the 367 strain tree and the 914 strain tree are nearly identical. For context, a Robinson-Foulds distance of 0 would indicate identical topologies, and the maximum Robinson-Foulds distance achievable in this scenario could have been 193. We have included discussion of this point in the methods section of the manuscript; specifically in the subsection titled *“Genetic and metabolic niche similarity”.*

2. Details of FBA regarding Objective function etc are missing from the Methods; a discussion on the choice of objective function for identifying flux distributions is important as in vivo objective functions may include more metabolic tasks than growth.

The objective function used in our FBA analysis was biomass synthesis. We believe that this is the best choice for an objective function in this context because we are using this analysis to ultimately predict genes that are essential for biomass synthesis. We have included this explanation in the methods, specifically in the section titled *“FBA and t-SNE dimensionality reduction/visualization”.*

Also unclear how flux variability was accounted for - 10 simulations are far too less for this. Was pFBA used?

We agree that 10 flux simulations is considered a small number of samples that cannot typically account for the total variation in flux states. However, due to the incredibly high dimensionality of our dataset it was computationally infeasible to produce 500 flux samples per GENRE and subsequently use a dimensionality reduction method. If we proceeded this way, 500 flux samples would produce a 457,000 samples x ~4,000 reactions data frame which is computationally expensive (infeasibly so) to reduce the

dimensionality via t-SNE. However, to ensure that we can capture sufficient variation in flux using only 10 samples per GENRE, we performed an analysis using a subset of 100 GENREs. We randomly selected 4 subsets of 100 GENREs and produced 100 flux samples per GENRE. We then performed the same t-SNE dimensionality reduction and visualization as presented in Figure 3. The outcome of this analysis is now presented in S3. We observed similar clustering patterns with this larger sample of fluxes (100), in each of the 4 randomly selected subsets of 100 GENREs. This result suggests that our original analysis using 10 flux samples per GENRE can capture variation in flux as well as an analysis that uses 100 flux samples. This analysis is now shown in S3. We have clarified and expanded upon this point in the manuscript, specifically in the methods subsection title "*FBA and t-SNE dimensionality reduction/visualization*".

3. Experimental validation: how were the four non-stomach bugs selected? Ideally a broader range of microbes would be useful to assess specificity of the targets. If less, at least some phylogenetic control should be applied.

Each of the four non-stomach isolates have important characteristics that make them great candidates for negative controls in this validation experiment:

1) Each of the non-stomach isolates are gram negative since each of the three stomach isolates are gram negative. This shared characteristic is important, because it means the presence/absence of a cell wall would not be a confounding factor when validating our computational predictions.
2) Each of the non-stomach isolates are from a different physiological location, which helps to support our hypothesis that the selected compounds were selective inhibitors of stomach-associated pathogens.
3) The four non-stomach isolates were from varying degrees of taxonomic relatedness to the stomach pathogens. One of the non-stomach pathogens (*P. gingivalis*) is from a different phylum as the stomach isolates, while the other three (*P. aeruginosa, E. coli, B. cenocepacia)* were from the same phylum. This variety in lineage also helps us to support our hypothesis that niche is a driver of metabolic function independent of taxonomy.
4) Finally, the selected isolates were readily accessible and easily culturable in our experimental system.

Further, we selected four non-stomach isolates for practical reasons; this was the number of isolates that we could feasibly test. Future work could expand the scope of this work and test additional pathobionts to take into account sufficient breadth/depth of taxonomy, gram positive/negative/variable, single/multiple isolates from all 28 other physiological locations, etc.

We have included these criteria in the manuscript in the results subsection "*Validation of stomach-specific pathobiont growth inhibitors*" to better explain the selection process for the non-stomach isolates used in the validation experiment.

4. Literature on conditional essentiality should be included in the discussion for giving a broader context to the study as it currently does assume an arbitrary complete growth media for all species.

We acknowledge that conditional essentiality is important in the context of our research because it is true that different media conditions will result in different essential gene predictions both *in silico* and *in vitro*. In our *in silico* simulations and *in vitro* validation experiments, we use a complete media. Essential gene predictions in a complete media context are more robust predictions because the more metabolites that are available in the environment for use, the more alternative metabolic pathways can be used by the organism, revealing which pathways are one-off, and truly essential. Thus, such predictions of gene essentiality in a rich media condition result in a minimal set of predicted essential genes. Alternatively, essential genes in a defined or minimal media produces a larger set of predicted essential genes. This idea was quantitatively explored in a previous study (Blazier and Papin, 2019); and showed that there is a linear relationship between the number of essential genes predicted with GENREs and the number of *in silico* media components. However, considering a media even more rich than LB, this linear relationship would eventually plateau due to limitations in the metabolic network. So if we were to identify a core set of condition-independent essential genes, we would need to define a media more rich than LB. However, we believe that the *in silico* rich media is sufficiently rich to capture a close-to-core set of essential genes. Overall, we can assume that the more components present in the *in silico* media, the fewer essential genes are predicted. By using a complete media in our simulations, we are left with a minimal set of essential genes that are maximally essential.

The *in silico* rich media formulation used in these analyses contains the following modelseed compounds: cpd00001 (Water), cpd00035 (L-Alanine), cpd00041(L-Aspartate), cpd00023 (L-Glutamate), cpd00119 (L-Histidine), cpd00107 (L-Leucine), cpd00060 (L-Methionine), cpd00161 (L-Threonine), cpd00069 (L-Tyrosine), cpd00084 (L-Cysteine), cpd00033 (Glycine), cpd00322 (L-Isoleucine acid), cpd00066 (L-Phenylalanine), cpd00054 (L-Serine), cpd00065 (L-Tryptophan), cpd00156 (L-Valine), cpd00220 (Riboflavin), cpd00644 (Pantothenate), cpd00393 (Folate), cpd00133 (Nicotinamide), cpd00263 (Pyridoxal), cpd00104 (Biotin), cpd00149 (Cobalt), cpd00971 (Sodium), cpd00099 (Chloride), cpd00205 (Potassium), cpd00009 (Phosphate), cpd00063 (Calcium), cpd00254 (Magnesium), cpd10515 (Iron), cpd00030 (Manganese), cpd00242 (Bicarbonate), cpd00226 (Hypoxanthine), cpd01242 (Thyminose), cpd00307 (Cytosine), cpd00092 (Uracil), cpd00117 (D-Alanine), cpd00067 (Hydrogen), cpd00567 (D-Proline), cpd00132 (L-Asparagine), cpd00210 (Taurine), cpd00320 (D-Aspartate), cpd03279 (Deoxyinosine) , cpd00246 (Inosine), cpd00311 (Guanosine), cpd00367 (Cytidine), cpd00277 (Deoxyguanosine), cpd00182 (Adenosine), cpd00654 (Deoxycytidine), cpd00412 (Deoxyuridine), cpd00438 (Deoxyadenosine), cpd00274 (Citrulline), cpd00186 (D-Glutamate), cpd00637 (D-Methionine), cpd00105 (D-Ribose), cpd00305 (Thiamin), cpd00309 (Xanthine), cpd00098 (Choline), cpd00207 (Guanine), cpd00082 (D-Fructose), cpd00129 (L-Proline).

We have updated the discussion to account for this point.

Reviewer #2:

The study by Glass et al. investigates evolutionary principles that may have shaped niche-specific metabolic functions in human bacterial pathogens, and applies these insights to identify antimicrobial targets specific to those niches. Utilizing genome-scale metabolic network reconstructions (GENREs) for known human bacterial pathogens from the BV-BRC database, the researchers identified physiological niche as a key factor in determining metabolic function. Through their in silico analysis, the researchers demonstrated the discovery of unique essential genes in stomach-associated pathobionts, and targeted these genes with small-molecule inhibitors. In vitro validation showed that inhibiting genes such as fabZ, fabF, and thyX selectively impeded the growth of stomach-specific pathobionts. This research highlights the potential of niche-specific antimicrobial strategies to mitigate the widespread effects of antibiotics and tackle the global antimicrobial resistance (AMR) crisis.

This work could be of great interest to the AMR community, as it advances our understanding of pathobiont metabolism and pioneers the development of targeted antimicrobial therapies based on metabolic and environmental specificities. The manuscript is well-crafted, and the display items effectively communicate the high quality of the research presented. However, to enhance the current version of the manuscript, I offer the following major and minor comments for consideration.

We thank the reviewer for the thoughtful comments.

Major comments:
1. A central theme of this work is the importance of niche-specific metabolic signatures identified through genome-scale metabolic pathway analysis. This approach, if successful, could mitigate the reliance on broad-spectrum antibiotics. However, there is a significant concern regarding the impact on commensal microbes. As the manuscript notes, phylogenetically distinct bacteria can exhibit similar metabolic functions due to convergent evolution. Therefore, *niche-specific* antimicrobials might still produce *broad-spectrum* effects within the localized physiological area, which is particularly concerning for gut microbes given their critical role in human health. This potential issue is the Achilles heel of the study and should be addressed in the manuscript. Additionally, I recommend that the authors conduct a proof-of-concept analysis to demonstrate that in silico knockouts of the three uniquely essential genes do not adversely affect the metabolic growth of a variety of stomach commensals.

We thank the reviewer for this important comment. We have added text to the discussion to address this important caveat which would need to be further developed in future related work and the potential application of this approach. Because of the vastly

large scope of commensal organisms, we did not consider the effect of our compounds on all commensal organisms *in silico* in this manuscript. In our specific use case (stomach pathogens), there are no native stomach commensals. However, we acknowledge that these compounds may influence commensal organisms or microbes in other locations, such as the member of the gut microbiome. To more directly address this point, we performed an additional *in silico* essential gene analysis to determine the presence of the identified uniquely essential genes (*mqo, ndk, aroE, cdh, fumC, tktA,* and *thyX*) in a select few commensal bacteria in the gut microbiome. Given the four most prevalent phyla of bacteria present in the gut microbiome (Firmicutes, Bacteroidetes, Proteobacteria, Actinobacteria), we randomly selected one species from each phylum (*Enterococcus faecium, Bacteroides intestinalis, Escherichia coli,* and *Bifidobacterium longum* respectively). We generated a GENRE of each of these species and performed an *in silico* single-gene-knockout analysis to generate a list of genes essential for producing biomass in an *in silico* complete media. Only one uniquely essential gene was identified as an essential gene in one of the commensal organisms. We have included a list of the essential genes for each of these four commensals in a supplemental table S6.

We acknowledge that this *in silico* knockout analysis in four commensal organisms is an incredibly small sample size to draw major conclusions about whether or not our identified uniquely essential genes would be present in all commensals in the gut microbiome or other microbiomes. However, it provides us with evidence that these identified uniquely essential genes are at least not ubiquitously essential in all gut commensals. In future work, we or others could more strongly consider commensal organisms by identifying all organisms considered to be human commensals, then generating a collection of all human commensal microbes, and finally use this new collection to support our computational predictions more strongly. We have updated the discussion section to acknowledge this critique.

2. The study emphasizes the role of environmental selection pressure as a driving force behind both divergent and convergent evolution. While the application of computational and empirical methods to explore these evolutionary dynamics is indeed innovative, the conceptual framework presented is not new. Evolutionary biology has long discussed these principles. Therefore, it is crucial that the authors more thoroughly integrate and discuss existing literature on this topic. Acknowledging prior foundational studies will provide a clearer backdrop against which the novelty of their own methodological and computational approaches can be distinguished. This could involve a more detailed comparison of their results with those of previous studies, particularly emphasizing any novel implications for antimicrobial therapy development.

We have expanded the manuscript to integrate existing knowledge about evolutionary biology concepts like divergent and convergent evolution of microbial pathogens. More specifically, we expanded the introduction to include a paragraph that discusses more existing literature on these topics, and clarification of how our research expands on these ideas in a new way.

3. The manuscript makes significant use of the MEMOTE score to assess the quality of metabolic network reconstructions, reporting an average score of 83%. For readers not familiar with genome-scale metabolic reconstruction, it would be beneficial if the authors could include a brief explanation of how the MEMOTE score is quantitatively defined. (This reviewer acknowledges the qualitative description in the Results section.)

We have included the following description of MEMOTE benchmarking scores in the manuscript for clarification:

"MEMOTE benchmarking ensures models follow typical community conventions, are in the correct SBML format, and are of high quality. In addition to model format, MEMOTE benchmarks models according to four primary areas: annotation, basic tests, biomass reaction, and stoichiometry. Annotation tests ensure that a model is annotated according to community standards: primary annotation identifiers belong to the same namespace and components are described using systems biology ontology terms. Basic model tests ensure that the model is formally correct: genes, reactions, and metabolites are present within a model. Biomass reaction tests ensure that biomass precursors are present and there is a resulting positive biomass when simulated *in silico.* Finally, stoichiometry tests ensure biochemical feasibility of the model; if inconsistency is identified, it is likely because of unbalanced reactions. A weighted score is generated for each of these four subcategories in addition to an overall MEMOTE score, which allows us to assess the quality of the model generated.

Clarification on whether an average score of 83% is indicative of high quality would be particularly helpful. Additionally, the assertion that this score is "…suggesting all reconstructions in the collection are of high quality…" is misleading. Given the variability inherent in any biological data set, it is likely there are some outliers with lower scores

We have clarified in the manuscript that an average MEMOTE score of 83% is high quality, and have supported this statement by referencing the MEMOTE score comparison analysis performed in Jenior et. al., 2023.

Further, we acknowledge that in any dataset there are outliers. To address this point, we have included the standard deviation and range in MEMOTE scores in our collection of models. Additionally we have included the average, standard deviation, median, maximum, and minimum for the overall MEMOTE score as well as the subcategories of consistency, metabolite annotation, gene annotation, and SBO annotation in a supplementary table S3.

4. The authors utilize 16s rRNA sequences to define genetic similarity among bacterial strains, limiting the analysis to 362 sequences out of the 914 strains due to the availability of 16s rRNA data. This methodological choice raises a concern about the comprehensiveness and accuracy of genetic similarity assessments within the study. Considering the availability of complete genome sequences for many microbial strains, using whole-genome data could provide a more robust and inclusive measure of genetic

similarity. Without asking to re-do the work, I recommend that the authors discuss the rationale behind relying solely on 16s rRNA sequences.

This is an understandable concern. We could not perform a multiple sequence alignment with whole genomes for 914 strains due to the computationally intensive nature of this analysis. As a solution, we decided to use only 16s rRNA sequences in an MSA. However, we acknowledge that a limitation of this approach is that we are constrained by the number of genomes that include 16s rRNA sequence annotations. To mitigate false positives and negatives that may appear in analyses because 367 species have 16s rRNA annotations, we took several precautions which are outlined in the response above to comment 1 from reviewer 1.

5. The statement that "these results underscore the potential of environment to shape unique metabolic function, and more importantly, show that the metabolic network reconstructions in our collection can capture complex evolutionary dynamics through constraint-based analysis" seems to overreach the conclusions that can be drawn from the data presented.

We have softened the language in this statement to more accurately reflect the role our collection of metabolic network reconstructions plays in this result.

The analysis described in Figure 2, which appears to rely on gene annotation analysis or gene functional enrichment, does not necessarily require the use of metabolic network reconstructions. Such analyses could potentially be conducted without the sophisticated framework of GENREs, using more straightforward bioinformatic tools. I suggest that the authors either clarify how the metabolic network reconstructions uniquely contribute to capturing complex evolutionary dynamics in ways that conventional gene annotation or enrichment analysis cannot, or consider revising the statement to more accurately reflect the methodology and its impact.

All analyses presented in Figure 2 do use GENREs. Figures 2a/b shows the distribution of reactions present across GENREs in our collection. While the reactions in GENREs *do* contain annotations that could be explored using other bioinformatics tools, the GENREs also contain reactions that were added during the gapfilling process. The gapfilling process adds reactions for which there is theoretical evidence (see gapfilling algorithm described in Jenior et. al., 2023), but that do not have explicit genetic/annotation evidence. So, figures 2a/b provide us with more information than annotated genomes alone would, because we are able to include gap-filled reactions in the analysis. We have added a statement in the results section "*Identifying metabolic reactions unique to subgroups of pathobionts*".

Secondly, figure 2c requires the use of our GENRE collection to generate the essential gene profiles (used for determining pairwise Metabolic Niche Similarity) for each pathobiont. The methods subsection titled "Genetic and metabolic similarity" contains information on how we used the GENREs to determine essential genes. We have

added language in the section “*Pathobiont metabolic niche analysis supports key evolutionary idea*s” to address this point.

6. The use of "flux samples" in the study raises several questions that are not addressed in the manuscript. Flux sampling is inherently dependent on the input conditions, such as the composition of the growth media. This dependency can significantly influence the metabolic state modeled by the GENREs. It is crucial for the authors to specify the media conditions used during these simulations, as this would impact the generalizability and applicability of the findings. Importantly, how do the authors know that their media conditions are physiologically relevant, especially in the case of the stomach?

We acknowledge that flux sampling is dependent on the *in silico* media context, and media context could have a significant impact on the results we see. We performed all of our *in silico* analyses in a complete/rich media context. We considered simulating each model on a defined media condition based on their physiological location, but we believed this would introduce the following issues in our analysis:

1) Our isolates are from 29 distinct physiological locations. While some of these physiological locations may have specific, defined, *in silico* media formulations that have been developed and rigorously tested, it is likely that some obscure physiological locations may not have sufficient specificity for the *in silico* formulations. Because of this potential difficulty, we wanted to ensure that the results of our computational analysis were not being influenced by the differences in *in silico* defined media. For example, in Figure 3, we would not have been sure that the differences we are seeing in clustering is due to the media condition, or the inherent metabolic differences between strains.
2) Compared to many defined media conditions, more alternative metabolic pathways are available in rich media conditions. Consequently, fewer genes are predicted as essential in rich media conditions, and those genes that are predicted to be essential are often more robust to changes across media conditions. This idea is more deeply discussed in the response to reviewer 1 comment 4.

We have included discussion of these points in the manuscript.

Additionally, the decision to use 10 flux samples per model rather than a single sample or another number should be justified.

We addressed this comment in our response above to comment 2 from reviewer 1

Moreover, it would be beneficial if the authors could explain how these 10 samples were differentiated from each other within the same model. Were there variations in the initial conditions, or were different constraints applied to each sample?

Metabolic network models are often underdetermined systems of equations which produce an infinite number of solutions. Rather than trying to find a single solution, we

perform flux sampling to analyze network properties. For flux sampling, we used an algorithm that was developed previously called GAPSPLIT (Keaty & Jensen, 2020). GAPSPLIT samples the model's solution space in an efficient way, with the goal of capturing the entire range of possible solutions. We have included this point in the text in the results section titled "*Pathobiont metabolic function is related to host physiological environment*".

7. Given that t-SNE includes a stochastic element and can sometimes exaggerate the identification of clusters depending on the chosen parameters, it would be useful for the authors to justify their preference for this method over more traditional and straightforward techniques such as Principal Coordinate Analysis (PCoA) or Multi-Dimensional Scaling (MDS). These alternatives are not only more commonly used in similar studies, but also tend to provide a more interpretable and reliable framework for understanding high-dimensional data. While the conclusions drawn by the authors may not heavily depend on the dimensionality reduction technique used, I am curious to see whether the same patterns and clusters observed using t-SNE would also be evident with these alternative approaches, which could further validate the findings and provide a more comprehensive understanding of the data.

We acknowledge that non-linear dimensionality reduction techniques like UMAP and t-SNE do not capture global relationships between datapoints; therefore, we take caution to not draw conclusions based on absolute distances between clusters. Instead, we consider our t-SNE analysis as a powerful qualitative visual that we can use to gain a deeper understanding of our data to inform biologically interesting and data-driven hypotheses.

Further, due to the high-dimensionality of our dataset, linear dimensionality reduction techniques like NMDS and PCA are not able to sufficiently display variation in the dataset. Because of this, the non-linear dimensionality reduction techniques were better for data visualization purposes and testable hypothesis generation, despite losing the ability to draw conclusions using absolute distances between clusters. To illustrate this point we have included S11, which displays the same data as Figure 3, but using NMDS and PCA (respectively) instead of t-SNE. We have included details of this analysis in the methods subsection "*FBA and t-SNE Dimensionality Reduction/Visualization".*

8. "Additionally, targeting to the infection site could reduce the need for bacterial community characterization since all organisms in the environment would be targeted." This echoes my point #1 above. Do the authors suggest that the eradication of all commensals in the niche might be acceptable, or is this an oversight? How does this strategy fundamentally differ from the problems associated with the use of broad-spectrum antibiotics, which also disrupt healthy microbial communities?

We thank the reviewer for this comment. We have changed the language in the manuscript to: "Additionally, targeting to the infection site could reduce the need for bacterial community characterization since all *pathobionts* in the environment would be targeted". We are not suggesting that the eradication of all commensals in the niche

would be acceptable, this is indeed an oversight. We hope that this work provides a first step in developing alternative antimicrobial therapies that do indeed mitigate the harmful effects of broad spectrum antibiotics. However, we agree that in future studies, developing a large collection of models of commensal organisms for cross-referencing our computational predictions and expanding our analyses would indeed strengthen our arguments. We have included this point in our discussion section.

9. It would be helpful to the reader if the authors could discuss limitations of the work and areas for future research, as is customary towards the end of the Discussion section in many high-profile papers.

We have significantly expanded our discussion section to acknowledge many of the limitations mentioned by the reviewers. We believe taking these comments into consideration has significantly strengthened the discussion section and the manuscript overall.

10. The definition of "unique reactions" used in the manuscript, set at a threshold where reactions present in 25% or fewer of the pathobionts are considered unique, appears overly inclusive. To this reviewer, reactions that are truly unique would be those found exclusively within a single class, genus, or species. Could the authors simply clarify whether any reactions met this stricter criterion of uniqueness? If so, detailing these findings could significantly strengthen the argument for the novelty and specificity of the metabolic functions uncovered in the study.

We selected this 25% cutoff criteria based upon a similar analysis that was performed in a previous study (Dillard et. al., 2023). However we have performed a supplemental analysis to determine if any reactions were present in only one strain, which we have mentioned in the manuscript and included in a supplementary figure (S7). We identified that there are 232 reactions unique to only one strain in the collection, which ensures that there are reactions that meet the strictest criteria for uniqueness. We have clarified and expanded on this point in the manuscript, specifically in the methods subsection titled "*Identifying core, accessory, and unique reactions and their corresponding metabolic subsystems*".

11. I found that there was a time delay in seeing the effects of alpha-mangostin on C. coli. Could the authors provide insights or hypotheses regarding this delayed response?

We would also like the reviewer to note that we do not claim that alpha-mangostin has an effect on the growth of *C. coli* based on these results. However, we notice that there may be a slight time delay of alpha-mangostin on *C. coli*. A possible explanation for this could be that alpha-mangostin does not affect the growth of *C. coli* in exponential phase, but does have an overall effect on maximum growth.

Minor comments:
1. Please add a space in "pathobionts(Figure 3d)". Also, please make sure that spaces are correctly placed before reference annotations, e.g., "for this application(29)".

We have addressed these comments in the manuscript.

2. Please remove the unnecessary space in "bacteria (28) ." (right before the period.)

We have addressed this comment in the manuscript.

3. In the Methods section, please change "taxid" to "taxID".

We have addressed this comment in the manuscript.

4. In the legend of Figure 1, please correct "1.15 metabolites" to "1.15 million metabolites".

We have addressed this comment in the Figure 1 caption.

5. The designations in the legend of Figure 1 are incorrect. For instance, "(d) The relationship between the number of genes and the number of reactions..." should be labeled as (g). Please revise accordingly.

We have addressed this comment in the Figure 1 caption.

6. Please correct the spelling of "uniuqley" in the caption for Table 1 to "uniquely".

We have addressed this comment in the Table 1 caption.

7. In the legend of Figure 2, it states the results in (b) are an enrichment analysis. This should be clarified as it is not a statistical enrichment but simply reporting proportions.

We have changed the words “Enrichment analysis” to “Relative proportions” to address this comment.

8. In Figure S1, the instruction "Select one strain per NCBI TaxID, strain with most pertinent metadata" is unclear. Consider revising the punctuation for clarity.

We have revised this statement in Figure S1.

9. For consistency, the title of Figure 3's legend should not be in title case. Also, add a space in "location(b)".

We have addressed this comment in the manuscript.

10. The legend for Figure 4 needs significantly more detail. For example, explain what the + and - symbols represent, the meaning behind the colors (green, yellow, orange) of these symbols, and how the confidence intervals (for the lines in the growth curves) are

defined. Additionally, change "α - mangostin" to "α-mangostin" (replace the en dash with a hyphen; remove unnecessary spaces).

We have significantly expanded the figure 4 caption to better explain the figure and address the above comment.

Reviewer #3:

The manuscript "Evolution shapes metabolic function and niche-specific antimicrobial targets in pathobionts" reports the development of constraint-based genome scale modelling approach to identify antimicrobial targets unique to specific microbial niches rather than to bacterial species. The authors construct and analyze the metabolic networks of >900 pathobionts (>300 species) and identify evolutionary principles guiding the development of unique niche-associated metabolic signatures. They further focus on stomach pathobionts, and identify and validate three genes unique essential to isolates of this particular physiological niche.

The topic of the manuscript is certainly relevant, the approach is innovative and the reconstructed network compendium is a valuable resource for the community. Therefore, I am generally enthusiastic about this story. However, I do have few issues that I think should be addressed in order to solidify the findings.

We thank the reviewer for the thoughtful comments.

Major comments

I think the manuscript would vastly benefit from streamlining to improve readability. In particular:

1. The subtitles within the results section do not always accurately reflect the findings. Or at least it is not obvious from the content of the text how to make it to the title. This also applies to the manuscript title. While the authors suggest evolutionary relationships throughout the manuscript, (not being an evolutionary biologist) in my view none of them is adequately tested/verified/substantiated in sufficient detail. The authors mostly suggest/predict such relationships. Thus, I advise caution in over-using them term "evolution", even in the tittle. Increasing accuracy of the title and subtitles towards what each section actually describes would likely solve the issue to a great extent.

We thank the reviewer for this comment. We have changed the title of the manuscript from “Evolution shapes metabolic function and niche-specific antimicrobial targets in pathobionts” to “Niche-specific metabolic function and associated antimicrobial targets in pathobionts”. Additionally, we have updated all subtitles in the manuscript to better reflect the contents of the section and removed the less-appropriate uses of the word evolution throughout the manuscript.

2. Explaining and interpreting the findings from Figs. 2c and 3a & b should be more precise, less speculative and better harmonized. The authors tend to very quickly dive into speculative interpretation, but do not provide accurate definitions of specific terms, neither description of general observations to be derived from the plots. The paragraphs below provide some specific examples (not exhaustive), as well as a few suggestions on how I think one could improve the issues.

We would like to thank the reviewer for helpful suggestions on how to improve the description of Figure 2 and Figure 3. We have used the comments below to improve the description of Fig 2c, and have also taken care to better explain and not so hastily dive into speculative interpretation in Fig 2c. Further, we have taken care to better connect and explain the ideas in Figure 3 and how they relate to the ideas presented while discussing the results of Fig 2c.

The section "Evolutionary drivers of unique metabolic function in pathobionts" and how it relates with Fig 2b is very difficult to follow. The message to be passed here is not clear/diluted among rather superficial/possible interpretations. I suggest a couple of possible improvements:

We have changed the title of this section from “Evolutionary drivers of unique metabolic function in pathobionts” to “Pathobiont metabolic niche analysis supports key evolutionary ideas”.

* Provide a more precise definition of "metabolic niche similarity". What is the meaning of "pairwise"? Improve the annotation of the axes in Fig 2c to overlap with text and ease readability of figure-text.

We have more thoroughly described “metabolic niche similarity” and “pairwise” in the text. We have changed the y axis title in Fig 2c to better match the text, and hopefully ease interpretability for the reader.

* The overall guidance for interpretation of Fig 2c could be facilitated by stating what is actually observed before trying to interpret it. For instance, what I first see from this plot is that similarity between essential genes is primarily higher than, and generally not correlated with, genetic similarity (assuming that I understand what is plotted). So it is somewhat unnatural that the first statement about this plot focus on "strong correlation". I total agree that this strong correlation is there - exceptionally for high similarity pathobionts, but it takes an eye to get there, especially if one is not familiar to the topic/representation.

We have added additional paragraphs prior to interpretation. We first added a paragraph to better describe the rationale for performing this analysis. Secondly, we described the definition of metabolic niche similarity and how we calculate this between pairs of pathobionts a bit more thoroughly. Third, we added a paragraph to describe the

observed logarithmic trend and how it is similar to a trend observed in a previously published study using commensals rather than pathobionts.

* While both variables don't seem to generally correlate, they do show a general trend, even well captured by line of best fit (which is otherwise not really used. Why get it then?).

We have decided to remove the equation for the line of best fit, since we agree that it does not add any valuable insight into our data. However, we have kept the trendline to aid the reader in visualizing the trend in the data and interpretation. Additionally, we have changed the way we refer to the data: we now note that there is a trend rather than a correlation.

While the authors equally highlight 4 different potential examples across the large cloud, it is unclear how they decided to subset these 4 different groups. Most importantly, the quantitative aspect that group 3 is clearly breaking (here I would dare say evolutionary) tendency, and potentially giving the most interesting outcome, is completely missed/not mentioned. To my understanding, this is the take-home-message of this plot: group 3 has a much higher pairwise essential gene similarity than what can be predicted from genetic similarity across the >900 GENREs. So I would be interested in knowing more about which pathobionts/genes are in fact grouping in this cloud - and whether they potentially have similar de facto metabolic niches, rather than a long list of examples sampling subsets 1, 2and 4 - which seem to follow the general trend anyway.

We believe it is important to discuss the groups now labeled as 3 and 4 because they are able to be described well with classical evolutionary concepts. Also, groups of pathobionts following these trends (3: genetically similar, similar metabolic niche; 4: genetically similar, difference in metabolic niche) will tie in nicely to trends observed in the data presented in figure 3.

We agree that the group of outlier pairs (now labeled as group 1) is quite interesting. Because of this observation we have taken care to more deeply investigate this group of pathobionts by identifying the members of group (genus/species), taxonomic class, and physiological location. Also, we now acknowledge the outlier group now labeled as 4 as well by identifying members, class, and location. We have included a supplementary figure to report this information on outliers.

* I would suggest a more descriptive tittle of this section. The current tittle does not really describe what is done/shown, and can mislead the reader towards the wrong expectation.

We have changed the title of this section from “Evolutionary drivers of unique metabolic function in pathobionts” to “Pathobiont metabolic niche analysis supports key evolutionary ideas”.

* The findings of this sections are disconnected from the remaining manuscript.

We have taken care to better connect the findings described in figure 2c with the subsequent figure 3.

Similarly to Figure 2c, the guidance provided for interpretation of Fig. 3 could be improved. For instance, the authors state "We observed several clusters of interest in these plots, which we considered more deeply for theoretical analysis." This might mislead the reader to think that both taxonomic class & physiological environment equally sustain the grouping of metabolic phenotypes. However, when looking at the figure, taxonomic cluster seems to be much more prominent than physiological environment - even though the second also seems to be there to a less extent. Also here, it would help streamlining the massage to make it more clear.

We have added additional context by describing Figure 3a and Figure 3b in a bit more detail before diving into the clusters of interest, to provide rationale for why we are interested in investigating individual clustering patterns more closely.

Important: what the authors mean with "in silico metabolic phenotypes" is again not clearly defined in the main text, and this hinders following interpretation.

We have defined the term “metabolic phenotype” in the introduction as “a unique functional metabolic state of a given organism determined by predicting the distribution of metabolic functions in the network.”

3. It is unclear how the authors selected the species/strains for testing drug activity.”

We addressed this point above in comment 3 from Reviewer 1.

The three non-stomach pathobionts are taxonomically so different from the stomach pathobionts, that one cannot be sure what actually drives drug activity - for instance drug transport or other intrinsic resistance mechanisms. I also miss the point the authors want to make: strain/species specificity or environmental niche specificity?

Three of the non-stomach pathobionts are part of the same phylum, while the fourth non-stomach pathobiont is from a different phylum. So while the non-stomach isolates are not from the same taxonomic class/genus as the stomach isolates, three of them are not entirely phylogenetically different.

We acknowledge that this validation analysis does not allow us to indicate the mechanistic driver of drug activity. To resolve this, we would need to more deeply profile each pathobiont and each drug; which is beyond the scope of this project. However, in light of this comment, we more closely looked into the literature regarding our selected inhibitory compounds to develop a better explanation of the results we produced. We have updated the manuscript to highlight these points and also point out in the discussion where there are discrepancies. We believe that although our analyses do not allow us to interpret mechanism of inhibition, this analysis could be valuable for

identifying which models may require more specific, targeted curation. Additionally, we are able to capture some potential off target effects of certain drugs that were reported previously in literature.

Additionally, we have made it more clear in the manuscript that the claim we are making is that the inhibitory compounds have environmental niche specificity.

We have expanded the discussion section to acknowledge these caveats.

Would it be more helpful to test taxonomically more similar strains (stomach and non-stomach) across different culturing medium - stomach-like vs non-stomach like? If possible. This would also support the manuscript towards establishing what is novel regarding the three selected drugs. Right now quite confusing and some text parts are redundant.

Similarly to what was stated as a response above to comment 6 from reviewer 2, we performed these validation experiments in complete media. We made this choice so that our computational *in silico* media conditions would be the same as our experimental media conditions. We considered using defined and physiologically relevant media conditions in both experimental and computational settings but believed this would introduce the following issues:

1) Our isolates are from 29 distinct physiological locations. While some of these physiological locations may have specifically defined *in silico* media formulations that have been developed and rigorously tested, it is likely that some of the more obscure physiological locations may not have such specificity in formulation. Because of this concern, we wanted to ensure that the results of our computational analysis were not being influenced by the differences in *in silico* defined media. For example, in Figure 3, we would not have been sure that the differences we are seeing in clustering is due to the media condition, or the inherent metabolic differences between strains.
2) Since we performed our in-silico simulations in complete media, we needed to also perform our computational validation experiments in complete media. If we used stomach-specific media in our validation experiment, there would be a chance we could not recapitulate our computational predictions due to the inherent differences we would be introducing into the system.
3) Model simulations, like essential gene predictions, are more rigorous in complete media conditions, compared to minimal/defined media conditions. For example, when predicting essential genes, a systematic single-gene-deletion method is employed. In a rich media condition, more alternative metabolic pathways are available to be used in a single-gene-deletion state. This decreases the probability that a gene is considered essential for growth, resulting in a minimal set of essential genes. This point is more deeply discussed in the response to reviewer 1, comment 4.

We have expanded our discussion section to acknowledge these points.

Minor comments
Abstract
Abstract could be a bit more streamlined. Some sentences - especially in the beginning - come unconnected. In addition, the third sentence doesn't read well - check grammar and revise.

We have revised the abstract.

Results section
Far stretch to understand what the author mean with "This logarithmic trend is consistent with the expectation that there are limited evolutionary advantages for bacteria with increasingly large genomes (17), which further validates the relevance of our collection." A more explicit explanation would be great.

We have changed this sentence to the following in the manuscript: *“As the number of genes in a pathobiont GENRE increases, the number of reactions in the GENRE increases up to a certain point. However, as the number of genes present continues to increase, there becomes a limit to the number of reactions present. This observation is consistent with the expectation that there are limited evolutionary advantages for bacteria with increasingly large genomes (17), and smaller bacterial genomes are known to have fitness benefits (18). The trend we see here further validates the relevance of our collection.”*

"We can analyze metabolic functionality through the generation of flux distributions feasible in a network reconstruction". This sentence doesn't read well - check grammar and revise.

We have changed the sentence to the following in the manuscript: *“We can analyze in silico metabolic phenotypes by generating flux distributions, which provide us with all feasible metabolic states in a GENRE”*.

I generally advice caution in using the word "enriched" and "significant" without a statistical test. For instance while describing the results of Fig 2b, both words are mentioned, but it is unclear to which null hypothesis the authors refer to.

We have removed the word “enrichment” from the manuscript and referred to this analysis as “increase in the proportion of reactions” to describe the type of analysis we are doing more directly. Further, we have removed the word significant from the manuscript since it was never used in the context of a statistical test.

Fig. 2:
Panel b could be re-ordered according to enrichment core vs unique to make it easier to find the different metabolic functions mentioned in the text.

We appreciate this suggestion from the reviewer. However, we have decided to keep the order of metabolic subsystems in figure 2b so the ordering is consistent with figure 2a.

Panels b and c: b and c are inside the plots. Looks strange.

We have move b and c to the outside of figures 2b and 2c
