# Supplementary material for: Niche-specific metabolic phenotypes can be used to identify antimicrobial targets in pathogens
Source: PLoS Biol. 2024 Nov 18;22(11):e3002907. doi: 10.1371/journal.pbio.3002907 (PMC11611258; doi:10.1371/journal.pbio.3002907)
Supplement: Attachment — Submitted filename: ResponseToReviewers.pdf [file pbio.3002907.s013.pdf]

You'll see that reviewer #1 says s/he's puzzled by your response to one of their previous queries, and emphasises some of the ways that it might be addressed. Reviewer #2 is very positive and just has some textual and presentational requests. Reviewer #3 is mostly positive, but has difficulties with your "Pathobiont metabolic niche analysis supports key evolutionary ideas" section, which s/he thinks is hard to follow and not well supported.

We appreciate the continued engagement of the reviewers and editor. We directly address each of these remaining concerns and in this revision. The review process has certainly improved the manuscript, and we are grateful for each of the comments & suggestions.

We realize that we misunderstood the original critique from Reviewer #1 regarding completeness and contamination. Consequently, our previous response was indeed puzzling. We have now directly addressed Reviewer #1's comment through a straightforward essential gene comparison analysis and added text to the discussion to communicate associated limitations.

We have addressed Reviewer #2's textual and presentational requests in the manuscript.

Finally, after some discussion, we agree with Reviewer #3 that the section "Pathobiont metabolic niche analysis supports key evolutionary ideas" section is not needed to support the rest of this manuscript. Although there are some interesting and thought-provoking ideas in this section that could be expanded upon in future projects, we have decided to remove the section from the present manuscript to not detract from key results and messages.

Reviewer #1:

I am puzzled by the authors' response to the completeness and contamination issue. The missing 16S comment was to highlight that essential genes can be missed. the "good quality" category used in genomic analyses have a different purpose and thus should not be used prima facie for essentiality analyses. I do recognise limitations in addressing this issue though but below are a few ways that I would like to suggest:

We now recognize that we misunderstood Reviewer #1's original critique leading to our confusing response in the previous revision. We have responded to this critique by (1) doing an additional analysis, and (2) addressing associated limitations in the Discussion section of the manuscript. Details to these points are provided below.

1. Select a subset of higher quality (say>95% complete) genomes and see if consistent results are found
2. Compare the results with known universally essential genes as +ve controls
3. add a discussion clearly identifying this limitation

The database we used to collected genome sequences (BV-BRC) does not have the option to filter by a completeness threshold. A user can either select sequences that are "good" (<10% contamination, >80% complete) or "not good" (>10% contamination, <80% complete). We selected "good" sequences for the analysis in our manuscript.

However, we did an additional analysis to illustrate that model predictions of essential genes of genomes with annotated 16S sequences (genomes that are likely more complete) are highly similar to the predicted essential genes of the entire 914 genome collection (which would include genomes that are potentially less complete).

With the presence of the 16S gene in the genome as a surrogate for a "more" complete genome, we shared results in the previous revision on the cladogram analysis for the "more" and "less" complete genomes. As reported previously, the underlying topologies of the cladograms are nearly identical, suggesting that the "more" and "less" complete genomes span similar microbial taxa.

While the cladogram analysis showed that both groups of genomes had similar coverage of microbial taxa, we performed an additional analysis with this revision to show that the functional predictions from both "more" and "less" complete genomes are also highly similar, suggesting that this completeness feature does not change the key messages from our analysis. We present in a supplemental document (S11) (also shown below) a histogram of the number of GENREs with a given essential gene across the subset of GENREs that are "more" complete (367 GENREs), and a histogram for essential genes across all GENREs (914 GENREs). Both are normalized to the number of members in the category. The similarity of the histograms suggests that the essential genes identified across the whole collection (914) is representative of the essential genes identified in more complete sequences (367).

Furthermore, the average difference in the presence of a given essential gene between the whole collection and the more complete subset is 1.3%. This result suggests that the essential genes identified in the entire collection of 914 GENREs (with "more" and "less" complete genomes) is representative of the more complete subset of GENREs (the 367 genomes that contain 16S sequences). This analysis helps to illustrate that the essential genes identified in the whole collection are similar to a subset with more complete genome sequences.

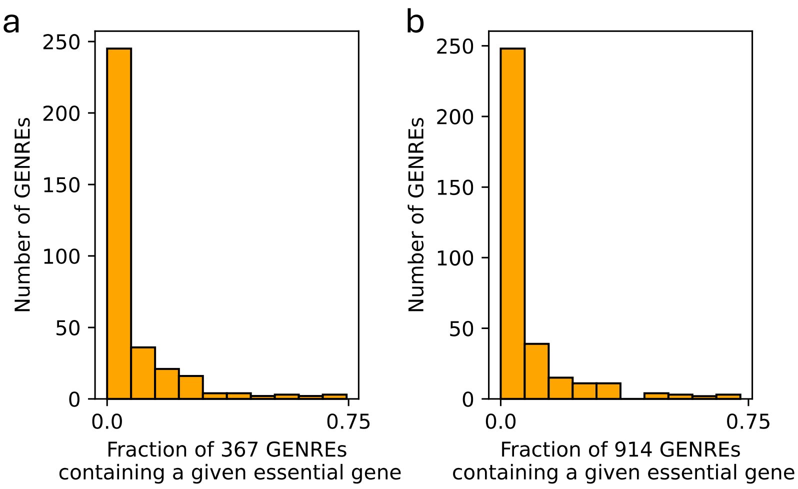

We included this analysis in a supplementary section S11.

To further address this critique, we have included mention of the limitation of completeness of our selected genomes in the discussion section.

Reviewer #2:

The authors have done a terrific job in addressing my extensive comments. I have no major comments to add at this point. I have only minor comments at this point to improve the readability of the manuscript.

Minor comments:

- Spacing before references remains inconsistent throughout the paper. Please see [1] and [2], for instance, and others.

We corrected the spacing before and after references throughout the manuscript.

- Figure 1c is not mentioned in the manuscript.

We included mention of 1c in the results section titled “Models of pathobiont metabolism”

- Please add a period after the last sentence in the legend of Figure 1.

We made this change in the Figure 1 caption.

- Is reference 23 on line 136 necessary?

This was an error and we removed it in the manuscript.

- "Consistent with previous studies, we observe clustering based on taxonomic class

[20,31,32] (Figure 3a)." It would be proper to put the references right after "previous studies" and not at the end of the sentence where you've described your finding.
We made this change in the manuscript.

- Line 311: "...organisms do not utilize. [35]." Please use one period for each sentence.
We made this change in the manuscript.

- Please see the legend for Figure 3. "Areas of interest from (a) and (b) are highlighted if (c) - (f) and described in the main text." This description is confusing to me. If c-f are described (and they are), then you've highlighted (a) and (b)?
We had a typo in this legend. Rather than "highlighted if", we meant to say "highlighted in". We have updated the manuscript and changed the word "highlighted" to "indicated" to try to be more clear.

- I apologize, but I am very confused by the structure of Table 1. What is the blank space on the lower right corner? Why are there eight genes in the table and not seven? Should fabF be in this table? It would be great if the co-authors could help make the table more visually appealing and better organized.
We apologize for the formatting of the table. We changed it to make it more understandable and to remove the white space in the bottom right corner. We also included *fabF* which was missing from this table.

- Line 360: Is there a reason thyX needs to be underlined?
This was an error. We have taken away the underline in the main text.

- The authors mention Figure 4a through 4u in the manuscript, but these indications are not present in the actual figure nor mentioned in the legend. Also, "i" and "j" are not mentioned in the main text.
We included an updated Figure 4 which includes panel labels. We edited the manuscript to mention 4i and 4j in the paragraph directly after the Figure 4 caption.

Reviewer #3:

First, I appreciate the efforts of the authors to satisfy the reviewer requests. Many of the requests have been addressed, especially to better document the quality of the dataset (e.g. contextualization of MEMOTE scores) and improve definitions used throughput the manuscript (e.g. metabolic niches). The precision and accuracy of the abstract, subtitles and general text has improved, and thus readability is better. I also appreciate the improvement and extra clarifications of the last section, and understand the limitations do expand on experimental efforts.

Nevertheless, in my opinion, the section "Pathobiont metabolic niche analysis supports key evolutionary ideas" remains very difficult to follow: difficult terminology, lack of precise examples from own data and poorly integrated in the rest of the manuscript. The authors

start with phenotypic evolution (which I don't really understand the meaning of), but never come back to that over the course of the section. While I appreciate the description of general tendency and clarification of the outlier group, many of my initial concerns remain: on what statistical basis do the authors distinguish sections 3 and 4 from the general tendency? Their interpretation of these two sections is based on general examples - none of the data that is actually at hand and part of the sections - and arguments and conclusions are made based on physiological locations, which are not part of this analysis and comes afterwards in figure 3. Also: Figure 2c is not mentioned early enough in the revised text, should be in line 206. Finally, the authors mention in section "The identification of uniquely essential genes informs stomach pathobiont-specific antimicrobial targets and growth inhibitors" starting at line 330: "To identify uniquely essential genes to stomach-associated pathobionts in silico, we first determined essential genes for all strains in the GENRE collection using an FBA single-gene-knockout method in COBRApy. Subsequently, we instantiated a universal essentiality threshold to determine uniquely essential genes across stomach-associated pathobionts (see Methods)." Why did they do this all again? Is it not what was done for Fig. 2c to determine metabolic niche similarity? It is my opinion that this section is particularly weak and, as is, not needed to support the rest of the manuscript.

After considering Reviewer #3's comments, we agree with the reviewer that the "Pathobiont metabolic niche analysis supports key evolutionary ideas" section is not well-supported and that it is not needed to support the key points of the manuscript. While we believe this analysis provides some interesting theoretical ideas that could be expanded upon in future work, we agree that it is not necessary to include in the present manuscript. We removed the section "Pathobiont metabolic niche analysis supports key evolutionary ideas" and Figure 2C from the manuscript.

Minor comments

Line 11: Pathobiont is used in abstract prior to definition.
We included the definition of pathobionts in the abstract as "organisms that have the potential to become pathogenic".

Line 57: Reference missing?
We added a reference to this sentence.

Lines 95-97: Sentence does not make sense. Suggest revise.
We revised this sentence for clarity.

Lines 125-127: While this attempts to give a reference to the reader, it comes out of context, and therefore does not really help. What is ModelSEED and CarveMe?
We edited this sentence for clarity. We explain now that ModelSEED and CarveME are two other automated GENRE creation tools often used in the field of metabolic network modeling. We added an additional sentence to this paragraph as well for clarification.

Line 144: I totally understand the concept of gapfilling, and thus I am aligned with the authors that it can add value to the networks beyond gene annotation limitations. However, this argument is vague/weak because it is not objective. How many reactions were added with gapfilling? Even if not many, are they essential reactions? Objectivity here would strengthen the argument.

We added a supplemental figure that shows a boxplot of the percent of total reactions/metabolites that are gapfilled across GENREs (Figure S4, also included below). We also stated in the text that on average, 13% of all reactions are gapfilled and 8% of metabolites are gapfilled across all 914 GENREs.

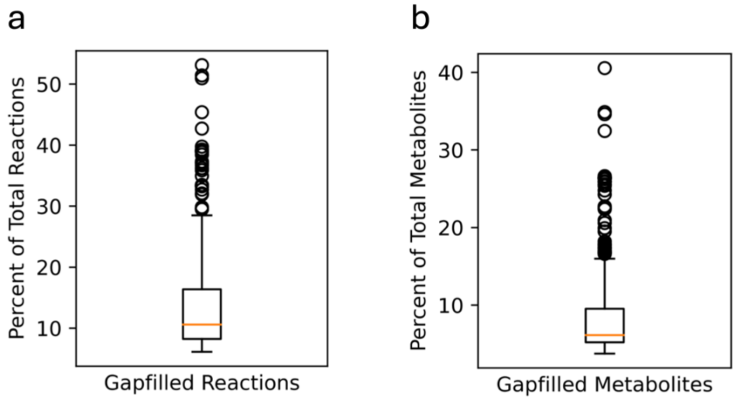

Gapfilled reactions are only added to the models to satisfy the criteria stated in the Reconstructor algorithm as described in the following reference (cited in the manuscript): Jenior ML, Glass EM, Papin JA. Reconstructor: a COBRApy compatible tool for automated genome-scale metabolic network reconstruction with parsimonious flux-based gapfilling. Bioinformatics. 2023;39. The criteria are that the metabolic network carries a minimum sum total flux and that constraints on biomass synthesis and other fluxes are satisfied. Consequently, all gapfilled reactions are essential to satisfy these criteria. Most, but not necessarily all, these gapfilled reactions are also essential for biomass synthesis.

Text was added to the manuscript to clarify this point in the Results section titled “Identifying metabolic reactions unique to subgroups of pathobionts”.

Lines 160 & 171: The start of these paragraphs is almost the same, but see to contradict each other:

"Most reactions across pathobionts were considered unique, which can be attributed to the large taxonomic range of pathobionts in the GENRE collection."

"The majority of reactions across pathobionts were considered unique to less than 25% of pathobionts showing that subgroups of pathobionts share these unique reactions"

Can the authors somehow illustrate what they want to say in the second paragraph? Otherwise it is difficult to follow.

We clarified the language in the second sentence so to better illustrate our point and resolve the so seeming contradiction.

We have changed the second sentence (originally at Line 171) to the following: "Interestingly, focusing only on the leftmost bin in Figure 2a, we observe that most of the metabolic reactions are present in less than 12.5% of pathobiont GENREs. This result suggests that there are certain subgroups of pathobionts that share these unique reactions, with the subgroups containing less than 114 pathobionts (12.5% of all pathobionts in our collection)."

Line 388: Respectfully, Gram-negatives generally have a cell wall!!! Gram-staining pertains to outer-membrane and not cell wall. The argument is correct though, because the outer-membrane is indeed a major barrier for antimicrobials in Gram-negatives. Please correct ;)
We thank the reviewer for catching this! We changed the text accordingly.

Discussion: Can/should be streamlined, as at places the language can be improved.

We edited the Discussion to streamline the text.
