# Supplementary material for: Niche-specific metabolic phenotypes can be used to identify antimicrobial targets in pathogens
Source: PLoS Biol. 2024 Nov 18;22(11):e3002907. doi: 10.1371/journal.pbio.3002907 (PMC11611258; doi:10.1371/journal.pbio.3002907)
Supplement: Attachment — Submitted filename: ResponseToReviewers.pdf [file pbio.3002907.s014.pdf]

IMPORTANT - Please attend to the following:

a) We wonder if you could make your Title more explicit and accessible (and declarative). Specifically, we suggest changing it to "Niche-specific metabolic phenotypes can be used to identify antimicrobial targets in pathobionts" - happy to discuss this by email.

We agree that the above title is clearer and more declarative, and have changed the title of the manuscript accordingly.

b) Relatedly, we're wondering how wedded you are to "pathobiont"? I note that one of the reviewers already asked you to define this, and I'm uncertain how widespread this term is. To what extent is your study aimed at *pathobionts* rather than *pathogens*?

After some discussion, we decided we are not wedded to the word “pathobiont”. We have changed all instances of the word pathobiont to “pathogen” in the manuscript. We have included a statement in the Methods sub-section “Genome-scale metabolic network reconstruction from genome sequences” to qualify our use of the word pathogen:

“For simplicity, we use the word pathogen throughout the manuscript to include bacterial species that are considered either pathogens or opportunistic pathogens.”

c) Please address my Data Policy requests below; specifically, we need you to supply the numerical values underlying Figs 1ADEFG, 2AB, 3ABCDEF, 4, S2, S4AB, S5 (treefile), S7, S8ABCDEFGH, 9ABCD, 10ABCD, and Figs 1 & 2 in File S11, either as a supplementary data file or as a permanent DOI’d deposition. I note that you already have an associated GitHub deposition, but this is currently only contains code and raw data. Please could you complete this deposition with the data underlying the Figures? IMPORTANT: Also, because Github depositions can be readily changed or deleted, please make a permanent DOI’d copy (e.g. in Zenodo) and provide this URL (see below).

We have provided all supplementary data underlying Figs 1ADEFG, 2AB, 3ABCDEF, 4, S2, S4AB, S5, S7, S8ABCDEFGH, 9ABCD, 10ABCD, and Figs 1 &2 in the Zenodo repository with the associated url https://zenodo.org/records/13952699.

d) Please cite the location of the data clearly in all relevant main and supplementary Figure legends, e.g. “The data underlying this Figure can be found in S1 Data” or “The data underlying this Figure can be found in https://zenodo.org/records/XXXXXXXX <https://zenodo.org/records/XXXXXXXX>

We have included this statement and URL in all main figure captions and supplementary figure captions.

e) I note that many of the supplementary files seem unnecessarily large, and should be cropped to reduce file size. "Figs" S3 and S6 would be better provided as Tables (spreadsheets) rather than these very large Tiffs.

We have cropped all Supplementary files and converted them to PNG files rather than Tiff files to significantly reduce the file sizes.

f) Please make any custom code available, either as a supplementary file or as part of your data deposition. I think this is probably already in your Github/Zenodo deposition, but I'm just checking.

We have added all custom code to Zenodo. We have updated our data availability statement with the Zenodo URL.

As you address these items, please take this last chance to review your reference list to ensure that it is complete and correct. If you have cited papers that have been retracted, please include the rationale for doing so in the manuscript text, or remove these references and replace them with relevant current references. Any changes to the reference list should be mentioned in the cover letter that accompanies your revised manuscript.

We have checked the reference list to ensure that no retracted papers have been cited.
